# Neurohormonal markers of stress in Borderline Personality Disorder: interplay between oxytocin and cortisol

**DOI:** 10.1192/j.eurpsy.2025.1933

**Published:** 2025-08-26

**Authors:** M. Teixeira De Almeida, L. Quattrocchi, N. Perroud, T. Aboulafia Brakha

**Affiliations:** 1 Department of Psychiatry, Geneva University Hospitals; 2 Faculty of Medicine, University of Geneva, Geneva, Switzerland

## Abstract

**Introduction:**

Borderline personality disorder (BPD) is characterized by important attachment issues and emotion dysregulation, particularly in response to psychosocial stressors. Emotions (mainly anger and sadness) may therefore be felt and expressed more intensively. Even though stress response is mainly regulated by cortisol, oxytocin is also involved in this response, and both hormones interact in a temporal dynamic. A previous pilot study carried-out by our group has shown that oxytocin displays less variation in naturalistic setting and lower reactivity during a stress task in patients with BPD when compared to healthy subjects.

**Objectives:**

We are now carrying out a larger-scale study to better understand the interplay between cortisol and oxytocin in every-day life and during stressful situations, but also which other factors may influence these hormonal changes like medication, hormonal contraceptives, childhood adverse events and other psychiatric comorbidities.

**Methods:**

We aim to recruit 120 participants (60 per group). Each participant undergoes three visits. First visit: explanation of the global procedure and risks, signature of informed consent, as well as psychological screening and distribution of questionnaires and salivettes (to be returned during the second visit). Second visit: return of questionnaires and return of part of the salivettes collected during the morning + additional saliva sample and assessment of emotional state. Third visit: experimental procedures and return of remaining samples of salivettes collected in the previous days for circadian assessment (the afternoon of the second visit). Thus, the day of the third visit participants perform an experimental task, the Trier Social Stress Test (TSST), involving stress reactivity.

**Results:**

Currently, 28 BPD patients and 39 healthy controls completed protocol. We analyzed hormonal preliminary data of the first 20 participants of each group. Our initial analyses showed a hyporeactivity of cortisol during stress in BPD patients compared with healthy subjects (F= 5.27, p = .005).

**Conclusions:**

Recruitment is still ongoing and further analyses will be conducted. Our study may offer a better understanding of the neurobiological underlying stress dysregulation in BPD, which may help to contribute to the development of new psychotherapeutic and pharmacological approaches.

**Disclosure of Interest:**

None Declared

